# Development of a Wearable Mouth Guard Device for Monitoring Teeth Clenching during Exercise

**DOI:** 10.3390/s21041503

**Published:** 2021-02-22

**Authors:** Rio Kinjo, Takahiro Wada, Hiroshi Churei, Takehiro Ohmi, Kairi Hayashi, Kazuyoshi Yagishita, Motohiro Uo, Toshiaki Ueno

**Affiliations:** 1 Department of Sports Medicine/Dentistry, Division of Public Health, Graduate School of Medical and Dental Sciences, Tokyo Medical and Dental University (TMDU), 1-5-45 Yushima, Bunkyo-ku, Tokyo 113-8549, Japan; r.kinjo.spmd@tmd.ac.jp (R.K.); chu.spmd@tmd.ac.jp (H.C.); k.hayashi.spmd@tmd.ac.jp (K.H.); t.ueno.spmd@tmd.ac.jp (T.U.); 2 Department of Advanced Biomaterials, Graduate School of Medical and Dental Sciences, Tokyo Medical and Dental University (TMDU), 1-5-45 Yushima, Bunkyo-ku, Tokyo 113-8549, Japan; uo.abm@tmd.ac.jp; 3 Clinical Center for Sports Medicine and Sports Dentistry, Medical Hospital, Tokyo Medical and Dental University (TMDU), 1-5-45 Yushima, Bunkyo-ku, Tokyo 113-8549, Japan; ohmi.spt@tmd.ac.jp (T.O.); yagishita.orth@tmd.ac.jp (K.Y.); 4 Department of Materials Engineering, Graduate School of Engineering, The University of Tokyo, 7-3-1, Hongo, Bunkyo-ku, Tokyo 113-8656, Japan

**Keywords:** teeth clenching, wearable sensor, mouth guards, sports performance, masseter muscle, electromyogram, force transducer, occlusal force

## Abstract

Teeth clenching during exercise is important for sports performance and health. Recently, several mouth guard (MG)-type wearable devices for exercise were studied because they do not disrupt the exercise. In this study, we developed a wearable MG device with force sensors on both sides of the maxillary first molars to monitor teeth clenching. The force sensor output increased linearly up to 70 N. In four simple occlusion tests, the trends exhibited by the outputs of the MG sensor were consistent with those of an electromyogram (EMG), and the MG device featured sufficient temporal resolution to measure the timing of teeth clenching. When the jaw moved, the MG sensor outputs depended on the sensor position. The MG sensor output from the teeth-grinding test agreed with the video-motion analysis results. It was comparatively difficult to use the EMG because it contained a significant noise level. Finally, the usefulness of the MG sensor was confirmed through an exercise tolerance test. This study indicated that the developed wearable MG device is useful for monitoring clenching timing and duration, and the degree of clenching during exercise, which can contribute to explaining the relationship between teeth clenching and sports performance.

## 1. Introduction

Dental occlusion may affect physical ability and body balance. Several studies have been conducted on the correlation between masticatory muscle or teeth clenching activity and sports performance [1,2,3,4,5,6,7,8,9,10,11,12,13,14,15,16,17,18,19,20,21,22]. A positive correlation between masticatory-muscle activity and performance in sports, such as volleyball, handball, soccer, and track and field, has been reported [1,19]. In weightlifting, it has been observed that teeth clenching and mandibular fixation improve performance [20]. The relationship between the occlusal force that accompanies exercise and muscle-power-exertion type has also been explained through an exercise tolerance test using the measurements of pressure-sensitive sheets [17,21]. To improve sports performance and health, it is important to investigate teeth clenching conditions during exercise.

With regard to static body balance against body sway during exercise and disturbance due to electrical stimulation, teeth clenching has been demonstrated to stabilize of the postural stance in several studies, which has been confirmed using an electromyograms (EMGs) of the masticatory and limb muscles, a force plate and an occlusal splint [3,6,9]. Regarding dynamic body balance, mandible movements during activities, such as hopping and running, evoke reflexes, thereby facilitating the active maintenance of the mandible posture. Additionally, the effects of teeth clenching with a mouth guard (MG) on masseter and sternocleidomastoid muscle activity were revealed using the EMG [8]. However, using a stabilometric platform, a prior study has reported that dental occlusion does not influence postural stability in static body balance [22]. There was no significant difference in dynamic body balance between pre-teeth clenching and post-teeth clenching interventions during jump-landing motions [11]. Some studies reported that an MG did not significantly affect the maximum exercise performance of athletes from a subjective point of view, such as numeric scores of the athletes’ assessments of mouth guard interference. Objectively, the maximum workload during spiroergometry and physiological parameters like oral airflow were also used to confirm this hypothesis [23,24]. The results of simple isometric exercise tests [25,26], such as the grip strength test, with respect to the relationship between dental occlusion and sports performance may disagree with those of complicated isotonic exercise tests, such as ball games, and opinions of athletes and healthcare professionals involved in the sports industry [27,28]. Therefore, experiments under practical conditions must be conducted to further explain the effect of dental occlusion on sports performance.

In previous studies [15], EMGs have been used to analyze the relationship between dental occlusion and sports performance. To record the EMGs of masseter-muscle activities, it is necessary to mount obstructive devices, such as electrodes and wires, on human skin. The EMGs may be affected by the unstable contact between the electrode and the human skin, and the electrode may become detached [29]. The use of wires is dangerous as they may get entangled with the body during exercise. It is difficult to determine the levels of occlusion and teeth clenching because EMGs measure muscle activity. Therefore, there is a need for a new wearable device that can measure teeth clenching through the force applied to the teeth during exercise.

Recently, wearable devices have attracted increased attention in sports- and healthcare- related research. Several studies have been performed on MG-type wearable devices, as listed in Table 1 [30,31,32,33,34,35]. Several studies have focused on the usefulness of an MG-type wearable sensors, including the acceleration sensor, developed for people suffering from concussions have been conducted [31,32,36]. Using the sensor during American football games, a study has been performed to detect the head impact [36]. In other research, an MG-type controller with pressure sensor and wireless data logger has been developed for tetraplegic patients [34]. These non-invasive MG-type wearable devices are advantageous in that they eliminate the danger and inconvenience of obtrusive external devices. Additionally, their position remained fixed in the mouth, and they can be easily worn and removed. Wearing and clenching an MG have been recommended in several sports to reduce the damage caused to the teeth, orofacial soft tissues, maxilla, and mandible at the moment of collision in contact sports such as rugby, boxing, and football [4,13,24,37,38].

This study aims to develop an MG-type device to monitor teeth clenching during exercise and demonstrate its potential. We have developed a wearable MG device, which consists of an MG with force sensors, to monitor teeth clenching during exercise. To evaluate the performance of the developed device, four simple occlusion tests—teeth clenching, teeth tapping, jaw movement, and teeth grinding tests—were performed. Subsequently, an exercise tolerance test was performed under practical sports conditions using an electromagnetically braked cycle ergometer. To our knowledge, this study represents the first attempt to use first preliminary data for monitoring teeth clenching during exercise using MG-type wearable device. We found that the developed wearable MG device was useful for monitoring the intensity, timing, and degree of teeth clenching during exercise.

## 2. Materials and Methods

### 2.1. Response of the Force Sensor in MG Materials

**Figure 1:**
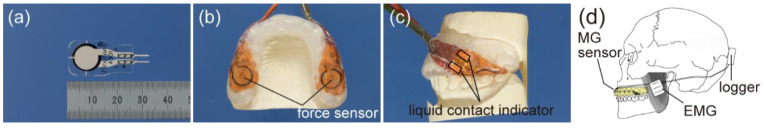
(**a**) Piezoresistive force sensor (FlexiForce); (**b**) occlusal surface; (**c**) side view of the mouth guard (MG) sensors; and (**d**) MG sensor and electromyogram (EMG) settings.

To confirm the response of the force sensor in MG materials, a piezoresistive force sensor (Flexi Force A301-25, Tekscan Inc., South Boston, MA, USA) (Figure 1a), which has been used in previous studies on occlusal force [30,32,39,40,41], and ethylene vinyl acetate (EVA) MG materials (ERKOFLEX, ERKODENT Inc., Pfalzgrafenweiler, Germany) were used in this study. The thickness of the force sensor is 0.203 mm; the sensor has a sensing-area diameter of 9.53 mm and can measure forces in the range of 0–111 N. A test sensor was developed by pressing the sensor between the EVA MG materials (thickness and diameter of 2 mm and 30 mm, respectively) at 90 °C using an electric hot plate and natural cooling.

The force sensor was placed on the platform of a universal test machine (EZ-LX, Shimadzu Co. Ltd., Tokyo, Japan) and vertically compressed by a rod (diameter of 8.0 mm). A load was applied on the force sensor using this machine at a speed of 0.25 mm/min. The sensor data were acquired using a logger (TSND151, ATR-Promotions, Kyoto, Japan) and an amplifier at 1000 Hz.

### 2.2. Fabrication of the MG Sensor

Three male participants (A: 29-year-old, B: 19-year-old, C: 28-year-old) volunteered for this study. They had normal dental occlusion, and they did not have any stomatognathic system disorder or any history of previous extreme injuries. This study was approved by the Ethical Committee for Human Research of the Faculty of Dentistry, Tokyo Medical and Dental University (no. D2018-060), and informed consent was obtained from the participants prior to conducting the experiment, conforming to the institutional guidelines.

To fabricate the MG sensors, dental stone models were prepared. After taking the dental impressions of upper and lower jaws with alginate impression materials (Aroma Fine Plus, GC, Tokyo, Japan), gypsum (Zo-Stone, Shimomura Gypsum, Saitama, Japan) was poured into the dental impressions. The dental plaster models were then completely cured and trimmed.

The MG sensor (Figure 1b,c) consisted of two layers of 2-mm EVA MG materials (ERKOFLEX, ERKODENT Inc., Pfalzgrafenweiler, Germany) and two force sensors (Flexi Force A301-25, Tekscan Inc., South Boston, MA, USA). First, the EVA MG sheet material was thermoformed over the maxillary dental plaster model using a vacuum-forming machine (Erkoform 3D plus, ERKODENT Inc., Pfalzgrafenweiler, Germany). The occlusal side of each maxillary first molar in the first layer was shaped to a flat surface to prevent distortion of the sensors when laminating the second layer. After being cut into the desired shape of the MG, two pairs of force sensors, which were covered by Kapton tape for insulation and waterproofing, were placed on the positions of the left (MGL) and right (MGR) sides of the maxillary first molars on the first layer (Figure 1b). Small liquid contact indicators were attached near the sensors to check whether moisture or liquid, such as saliva, leaked into the device (Figure 1c). Subsequently, the second layer of EVA MG sheet material was laminated over the MG with sensors using the same vacuum-forming machine. The occlusal surface was added to the second layer at the intercuspal position using the mandibular dental plaster model. The thickness of the occlusal surface of the maxillary first molar was approximately 2.5 mm. After natural cooling, the wearable MG sensor device was trimmed and finished with dental bars and a buffing wheel. A second layer corresponding to the terminals was notched and the lead wires were connected to the sensor terminals and carefully sealed using a heating gun.

### 2.3. Dental Occlusion Test with the MG Sensor

The following four types of dental-occlusion tests were performed with the MG sensor: teeth clenching, teeth tapping, jaw movement, and teeth grinding. At the beginning of each test, 100% maximum voluntary teeth clenching was conducted, and all subsequent data were normalized by the initial data.

During the dental-occlusion tests, the participants were seated on chairs and at rest. The MG sensor was attached to the participant and the logger (TSND151, ATR-Promotions, Kyoto, Japan) was connected to the MG sensor. The EMGs were simultaneously recorded from both sides of the masseter muscles using a wireless multi-channel digital telemetry system (WEB-1000, NIHON KOHDEN Corporation, Tokyo, Japan) at 1000 Hz, which included a band-pass filter (20–500 Hz), on a personal computer (CC-700H, NIHON KOHDEN Corporation, Tokyo, Japan) with a receiver (ZR-100H, NIHON KOHDEN Corporation, Tokyo, Japan). After adequate preparation of each participant’s skin, including shaving and cleaning with alcohol and cotton, the electrodes (ZB-150H, NIHON KOHDEN Corporation, Tokyo, Japan) were attached to the skin over the center of the masseter muscles on the left (EMGL) and right (EMGR) sides at the midpoint between the anterior end of the zygoma and the retromandibular portion. The inter-electrode distance equaled 3 mm.

The movement of the mandible was recorded using a camera (iPhone XS Max, Apple Inc., California, USA) at 240 fps; to this end, a position marker was placed on the skin of the mandibular midline. The video was analyzed using motion analysis software (Kinovea version 0.8.27). The details of the dental-occlusion tests are as follows.

1. Teeth clenching: teeth clenching, performed for 5 s at four different clenching intensity levels from 100% to 25% in steps of 25%, and light teeth touches were examined using visual feedback control to determine the relationship between the sensitivity of the MG sensor and the EMG. The participants were instructed to match each intensity level of the MG sensor output displayed on a monitor in front of them.
2. Teeth tapping: strong and quick teeth-tapping tasks were examined for approximately 10 s to compare the temporal resolutions of both sides of the force sensors in the MG sensor and EMGs using the peak interval time and the full width at half maximum (FWHM).
3. Jaw movement: first, maximum voluntary teeth clenching was performed at the intercuspal position. After holding at the resting position, left lateral movement of the mandible, right lateral movement of the mandible, and movement of the lower jaw to the protruded position, teeth clenching was performed to check the responses of the left and right force sensors when the mandible moved from side to side and forward. Each motion required 5 s, and there was a 5-s interval between the different motions.
4. Teeth grinding: the participants ground their teeth laterally for approximately 10 s; the EMG was compared with the MG sensor output obtained from teeth grinding.

The MG sensor and EMG data were analyzed using scientific graphing and data analysis software (Origin, version 2019b, OriginLab Corporation, Northampton, MA, USA). EMG data were calculated by the root mean square (RMS, smoothing time = 50 ms) conversion of raw EMG data. Each EMG data point was divided by the amplitude at each time of maximum muscle exertion (%MVC; maximum voluntary contraction) and then normalized. The data were statistically analyzed using one-way analysis of variance with the Tukey–Kramer honestly significant difference test (α = 0.05) using statistical analysis software (JMP, version 13, SAS Institute Inc., Cary, NC, USA).

### 2.4. Demonstration Measurement during Exercise

An exercise tolerance test was performed using an electromagnetically braked cycle ergometer (Power Max VIII, Konami Corporation, Tokyo, Japan) to examine the usefulness of the MG sensor in monitoring teeth clenching during exercise and sporting activities. After performing a 5-min bicycle warm-up, the participants were required to perform the exercise tolerance test, wherein they pedaled for 10 s at their maximum power. The test had three stages of load levels, which were separated by 2-min rest periods. The load setting for the first stage was determined based on gender and weight (3.0 kilopond [kp]). The built-in computer program automatically set the load value for the second stage (4.0 kp) according to the speed of the first stage. The load value of the third stage was also set automatically to 5.0 kp, which was the maximum load. The foot motion was tracked using a camera (iPhone XS Max, Apple Inc.) at 240 fps and motion-analysis software (Kinovea version 0.8.27).

## 3. Results

### 3.1. Sensitivity of the Force Sensor in MG Materials

**Figure 2:**
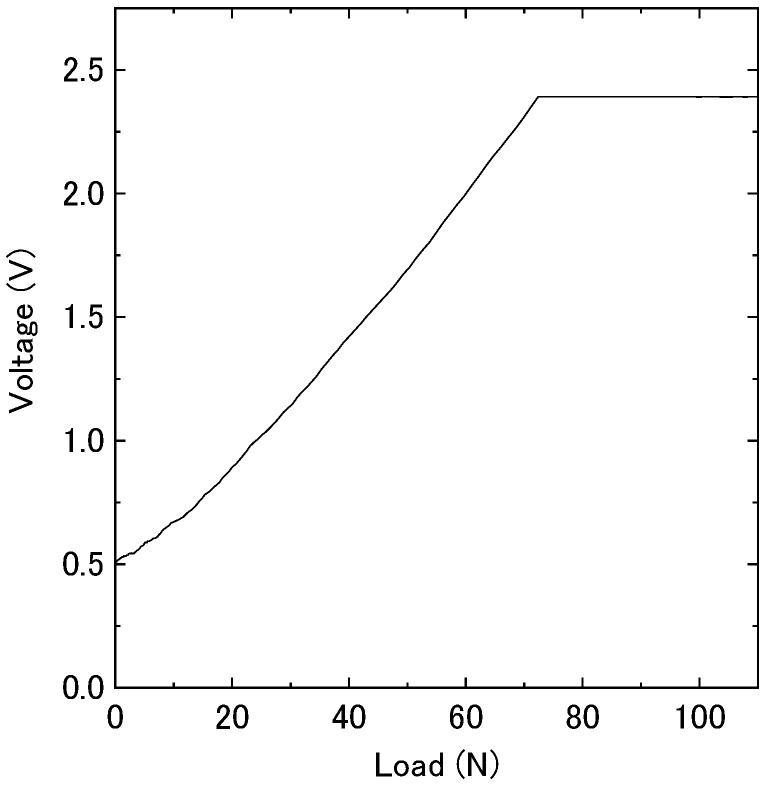
Correlation between the output voltage of the force sensor in MG materials and the load.

Figure 2 shows the correlation between the output of the force sensors in the MG materials and the load when applying the load to the force sensor. The rise in force sensor output voltage of the force sensor was linear up to a load of 70 N. In this setup, forces over 70 N cannot be reliably measured unless the setup, especially amp, is adjusted.

### 3.2. MG Sensor Response to the Dental Occlusion Test

During the experiment, the color of the water sheet-type indicator inside the MG sensor did not change, indicating that a liquid, such as saliva, was absent near the force sensors.

**Figure 3:**
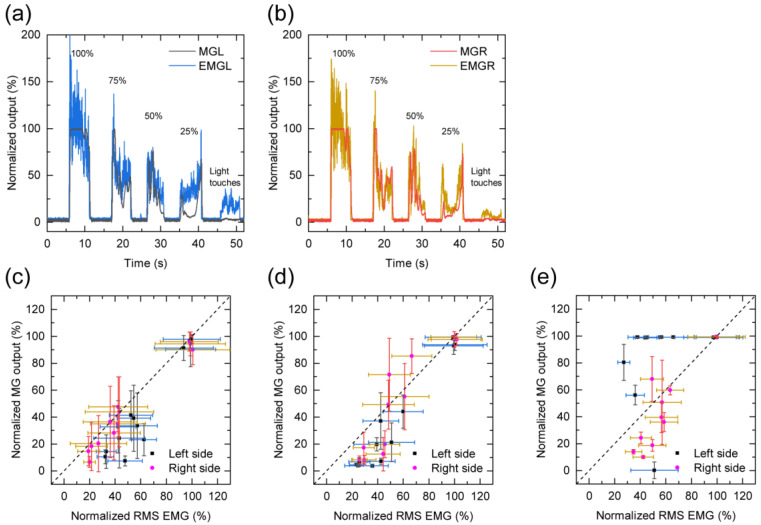
MG sensor outputs and the electromyograms (EMGs) during the teeth-clenching task at four different levels from 100% to 25% in steps of 25% and during light teeth touches. (**a**) Left-side sensors (MGL and EMGL); (**b**) right-side sensors (MGR and EMGR); and correlation between the MG sensor output and EMG (right and left sides, respectively) for each participant: (**c**) participant A, (**d**) participant B, and (**e**) participant C. Error bars represent the standard deviation.

Each MG sensor output under maximum voluntary teeth clenching was approximately 2.4 V, and it was used to normalize each MG sensor data. The teeth-clenching task results are presented in Figure 3. The MG sensor noise was low compared with that of the EMG (Figure 3a,b), and the timing of teeth clenching could be clearly detected. The relationship between the MG sensor and EMG is illustrated in Figure 3c. The MGL output of participant C deviated from a linear proportional relationship. The standard deviation of the MG sensor was similar to that of the EMG except at the time of maximum voluntary clenching.

**Figure 4:**
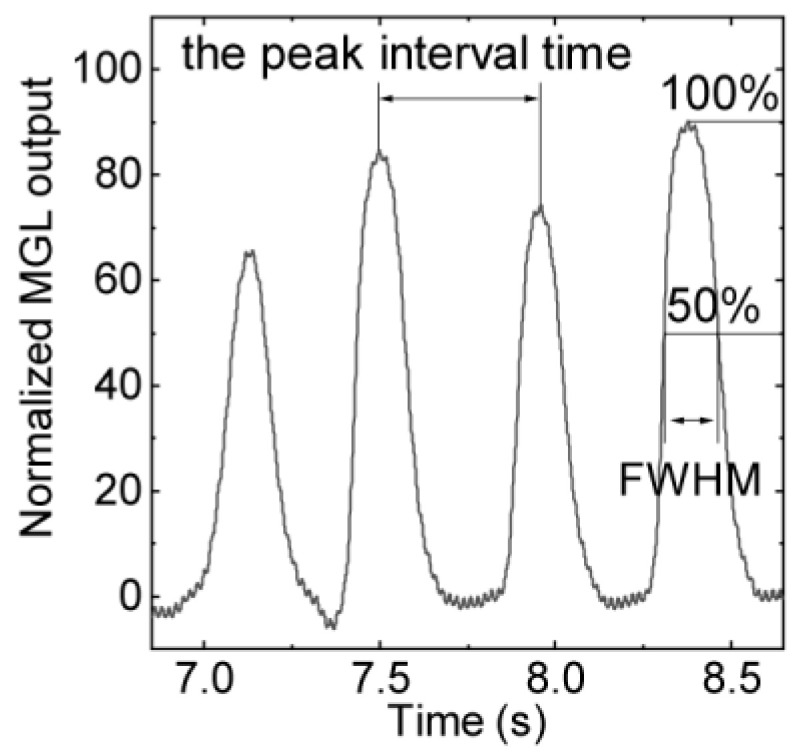
MG sensor output during the teeth-tapping task.

In the teeth-tapping task, the peak interval times of the MG and EMG sensors (Figure 4) were not significantly different (*p* > 0.05), as shown in Table 2. These participants performed 3.5 (A), 3.3 (B), and 3.2 (C) teeth taps per second. In addition, in Table 2 the FWHM of the MG and EMG, except the MG of participant C, indicates that there were no significant differences between the left-side and right-side sensors (*p* > 0.05), but there was a significant difference between the MG sensor output and EMG (*p* < 0.05). The FWHM of the MG sensor was statistically larger than that of the EMG (*p* < 0.05).

**Figure 5:**
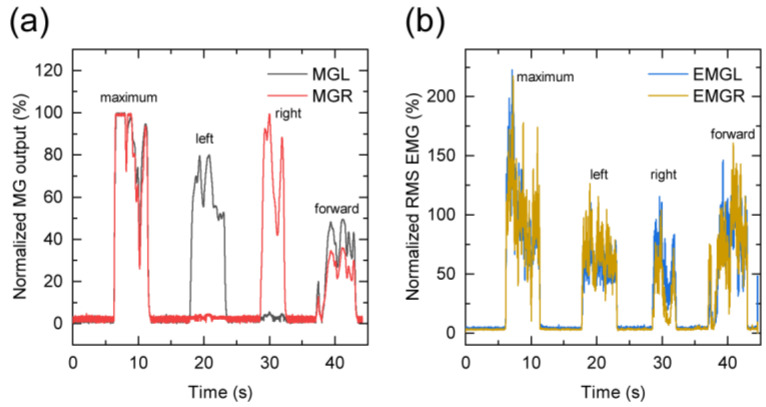
(**a**) MG sensor output and (**b**) EMG during the jaw movement task. These four peaks indicate the maximum teeth voluntary clenching and the left lateral, right lateral, and forward movements of the mandible, respectively.

In the jaw movement task, the first, second, third, and fourth wave-forms indicate the maximum voluntary teeth clenching, left lateral movement of the mandible, right lateral movement of the mandible, and forward movement of the mandible respectively (Figure 5a,b). The results of each movement are summarized in Table 3. With regard to participant A, when the mandible moved laterally to the left, the MGL output demonstrated an increase compared with the MGR output. Similarly, when the mandible moved laterally to the right, the MGR output exceeded the MGL output. The MGL output is similar to the MGR output when moving forward. Regarding participant B, the MGR output increased in comparison to the MGL output when the mandible moved laterally to the left and right and moved forward. In the case of participant C, the MGL output exceeded the MGR output when moving forward. EMG activity is shown when the mandible moved laterally to the left and right and moved forward in all participants.

**Figure 6:**
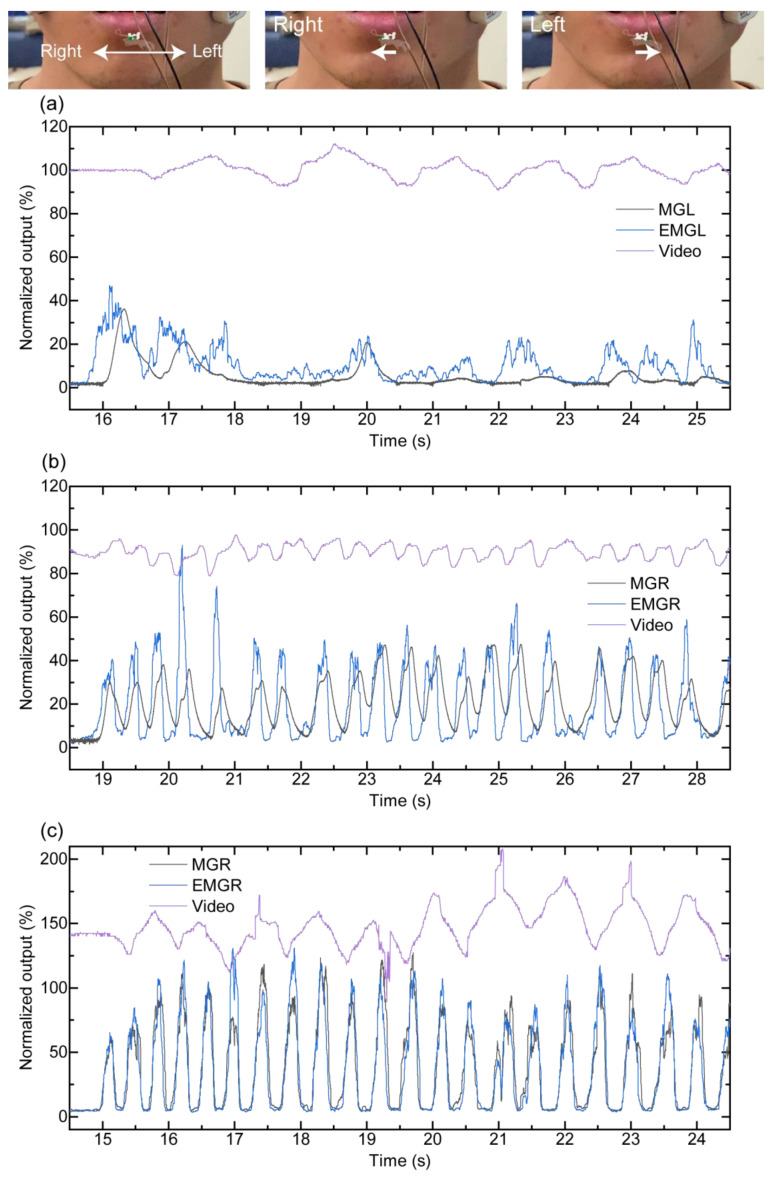
Sensors compared with the video-motion analysis during the grinding task for each participant: (**a**) participant A, (**b**) participant B, and (**c**) participant C. Video analysis shows how the median of the mandible moves to the left and right sides with respect to the median of the maxilla.

In the teeth-grinding task (Figure 6), the video-motion analysis result confirmed that the mandible moved to the left and right repeatedly. There were approximately 0.8(A), 2.2(B), and 1.3(C) teeth grinds per second. The MG sensor output peaks are shown clearer than those of the EMG. In addition, EMG peaks when the mandible moved laterally to the left and right quickly are also shown. With regard to participant A, the video-motion analysis results agree with the peaks of the MG sensor. Regarding participant B, both the MGL and MGR outputs increased when the mandible was on the left side according to the video-motion analysis. The peaks of both sides of the EMG are shown while the mandible moved from the right side to the left side. For participant C, both the MGL and MGR outputs increased during the mandible movement from side-to-side, and these outputs decreased when the mandible moved laterally to the left and right, according to the video-motion analysis. In addition, both sides of EMG increased when the mandible was on the left side and right side.

### 3.3. Exercise Tolerance Test with MG Sensor

**Figure 7:**
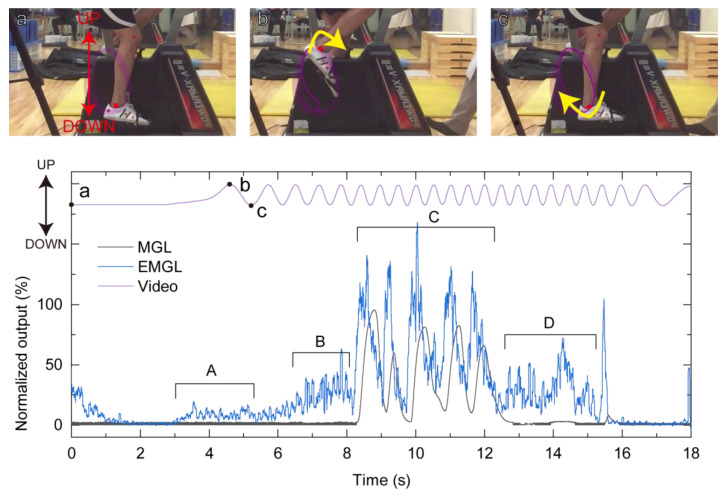
Outputs of left-side sensors (MGL and EMGL) compared with the video-motion analysis during the exercise tolerance test. Although EMGL shows approximately 5–40% output during periods A, B, and D, the MGL output shows almost 0%. This means that the participant did not clench the teeth even when masseter muscle activity was detected. In periods C, both MGL and EMGL indicate high levels, around 100%, meaning the participant clenched the teeth.

Figure 7 shows the output of the left-side sensors (MGL and EMGL) and video-motion analysis during the second stage of the exercise tolerance test of participant A as an example. The outputs of the left and right sides of both the force sensors in the MG sensor and EMG were similar. Although the EMG exhibited a low level (5–30%) at 3.0–5.0 s (A period), 6.0–8.0 s (B period), and 12.5–15.0 s (D period) from the start of the test, the MGL output was almost 0% during this time. This implies that the participant did not clench his teeth even though an electric-field change was detected. At 8.5–12.5 s (C period), the MGL output and EMGL were almost 100%; this suggests that the participant clenched his teeth five times at a high level of approximately 100%.

## 4. Discussion

In the teeth-clenching task, the MG sensor output exhibited less noise compared with that of the EMG. This was a result of differences in the measurement mechanism of the MG sensor and EMG. EMGs can detect a weak electric-field change. However, because EMG signals are highly susceptible to the introduction of noise, the signal to noise ratio remained low. In contrast, the piezoresistive force sensor observes the resistance change caused by the force applied to the sensor and an appropriate S/N ratio can be obtained by selecting a sensor material with a suitable measurement range. Generally, when examining the relationship between muscle activity and occlusal force in an individual, the greater the amount of muscle activity, the greater the occlusal force generated [42,43,44]. This is consistent with the finding in this study that a larger %MVC facilitates attainment of a higher sensor output (Figure 2). The MGL output of participant C was higher than that of the others (Table 1). It is believed that the MGL output of participant C was higher because participant C had a large occlusal contact area on the left side with different teeth alignment and individual tooth structure. Toma et al. [34] reported on occlusion force measurements taken with an MG controller, including two pairs of pressure sensors, using a jaw phantom. This report [34] suggested that a calibration curve for each sensor position is required to improve the measurement accuracy.

In the teeth-tapping task, the peak interval times of both sides of the force sensors in the MG sensor and EMGs had no statistical difference. The ability to measure 3.5 teeth taps per second demonstrated that the MG sensor possessed sufficient temporal resolution for human occlusion. It is believed that the MG sensor has an equivalent sensitivity and specificity as the EMG. In the jaw movement task, force sensors in the MG sensor reacted according to mandible deviations. In general, the MGL output must increase compared with the MGR output when the mandible moves laterally to the left. Similarly, when the mandible moves laterally to the right, the MGR output must increase, compared with the MGL output. When the mandible moves forward, the MGL output and MGR output must increase at the same level. Although the results of participant A showed these results, the results of participant B were different. Regarding participant B, the MGR output increased compared with MGL output when the mandible moved laterally to the left, to the right, and forward. These results show the potential of the proposed MG sensor to monitor the lateral jaw deviation. This is a result of the teeth alignment and arrangement of the force sensors and is a useful clue in determining the forward deviation of the jaw. In addition, it may be possible to confirm the deviation of the jaw by examining the balance between the outputs of the left and right sensors. In the teeth-grinding task, in regards to the MG sensor, we found that large and small waves were repeated alternately on both the left and right sides. Compared with the video-motion analysis, the peaks of the MG sensor were shown when the mandible moved laterally and were at the left and right sides. It was found that the MG sensor could be influenced by the teeth alignment, arrangement of the force sensors, and limitation of the temporomandibular joint movement. Some peaks became unclear because there was a problem with contact of the EMG with participants’ skin during the experiments. Therefore, compared with the EMG, the MG sensor can provide more information about the occlusal contact state and physical damage to the teeth. The MG sensor can be useful for research on bruxism during sleep and sports that cause lateral jaw movement.

During most of the exercise tolerance test, the MG sensor output exhibited minimal reaction. This means that the participant did not clench during most parts of the test. At 8.5–12.5 s (Figure 7, C period), the MG sensor output and EMG exhibited a value of nearly 100%, indicating teeth clenching. Hoshino et al. [17,21] reported on the occlusal force of teeth clenching during the exercise tolerance test using the pressure-sensitive sheets. Although the total occlusal force of the participants could be measured with the pressure-sensitive sheet, clenching timing and duration during exercise were not fully explained. In this study, it was found that clenching timing and duration during the exercise tolerance test, as well degree of teeth clenching, were comparable to that at maximum occlusion using the MG sensor. These results provide scope for further discussion of the relationship between teeth clenching and sports performance in complicated motion sports. In terms of EMG activity, many peaks, which were not detected by the MG sensor, were detected, especially at times of approximately 3.0–5.0 s (Figure 7, A period), 6.0–8.0 s (Figure 7, B period), and 12.5–15.0 s (Figure 7, D period). The effect of the sensor motion on the muscle activity measurement has been reported [45,46]. Akio Himejima et al. [47] reported an increase in the digastricus muscle activity even when both the occlusal contact and amount of test muscle activity were not similar to maximum clenching during judo activity. This means that the EMG activity could increase without clenching during exercise. It is difficult to confirm using EMG results alone that teeth clenching did not cause these peaks. By combining the MG sensor and EMG results, it is believed that EMG artifacts caused these peaks, or that the masseter muscle activity appeared without teeth contact.

This study indicates that the MG sensor is a useful tool to determine teeth clenching tendencies during exercise. To the best our knowledge, compared to conventional devices, the MG sensor fabricated in this study represents the first attempt to monitor teeth clenching during exercise. The proposed device can be employed in research concerning teeth clenching not only during exercise but also in other contexts, such as sleep bruxism and temporomandibular disorders. However, this study has certain limitations. Because this study primarily aimed to confirm the potential utility of the MG sensors, the exercise tests performed were preliminary in nature. Real-world sports scenarios require consideration of several complex factors, such as different complicated motions, assigned roles of different players, and the type of the sport. These factors considerably affect the relationship between teeth clenching and sports performance. Therefore, the results of the exercise tests presented here are insufficient to discuss the effect of clenching on sports performance. In future research, with due consideration of these factors, we intend to perform experiments with and without clenched teeth, as well as with and without a mouth guard to determine the effect of clenching. This would help us better explain the relationship between teeth clenching and sports performance, thereby facilitating improvement in the dental health and performance of athletes.

## 5. Conclusions

In this study, an MG sensor was fabricated and characterized using a dental-occlusion test. Additionally, occlusion measurement during exercise while wearing the MG sensor was demonstrated. We found that the MG sensor has a good capability to detect occlusal forces as well as a sufficient temporal resolution. The MG sensor response depends on the arrangement of sensors and occlusion, which may lead to the determination of occlusion using MG sensor data. In occlusion measurement during exercise, the clenching timing and duration as well as the degree of teeth clenching were determined using the MG sensor. These results reveal that the MG sensor is a useful tool for determining personal teeth clenching tendencies during exercise. In the future, it may be possible to understand these tendencies depending on the skill level by performing relevant experiments.

## Figures and Tables

**Table 1:** Prior study focusing on mouth guard type sensors to measure physical quantities (force and acceleration). It is difficult to compare the force-measurement ranges considered in these studies because there are different measurement methods, e.g., force was applied to force sensor and mouth guard (MG)-type sensor.

	Sensor Type	Objective	Performances
Diaz et al. (2012) [30]	Force sensor	Monitoring of human bruxism	Bruxism events in vitro trials were performed for validation Force sensor (ZFLEX A201-100, Tekscan Inc., South Boston, MA, USA) was used. Measurement range: 0–about 1000 N, Sampling rate: unknown, in vitro and in vivo tests
Camarillo et al. (2013) [31]	Acceleration sensor	Measuring 6-DOF head kinematic response during impact	Laboratory-based impact testing performed at points on the helmet and facemask
Kuo et al. (2016) [32]	Acceleration sensor (and force sensor)	To evaluate the mandible constraint’s effect on MG’s kinematic measurement accuracy	Free fall drop experiments performed on football helmeted ATD * and PMHS * heads over a range of impact locations and heights while varying the mandible constraint Force sensor (FlexiForce A201, Tekscan Inc., South Boston, MA, USA) was used to check mandible constraint, Measurement range: −300 N, Sampling rate: unknown, in vitro test
Toma et al. (2018) [34]	Force sensor	Expecting that oral motion can be used to control external devices because occlusion or tongue motion remains possible for tetraplegics	Measuring occlusal pressures at three different positions—front tooth and right and left second molars—in an MG-type controller with pressure sensors Measurement range 1.7–50.2 N, Sampling rate: unknown (data from a logger were transmitted with a time interval or 30 s.), in vitro test
Wu et al. (2018) [36]	Acceleration sensor	For detecting field football head impacts	Validating instrumented MG data from collegiate football games and practices, with ground truth data established from video review
Proposed study	Force sensor	For monitoring timing, duration, and a degree of teeth clenching during exercise to explain the relationship between teeth clenching and sports performance	Four types of dental occlusion tests -clenching, tapping, jaw movement and grinding- and an exercise tolerance test with an MG sensor device Measurement range: 0–100 N, Sampling rate: 1000 Hz, in vivo test

* ATD: anthropomorphic test dummy; * PMHS: post mortem human surrogate.

**Table 2:** The mean of peak interval time and the mean of full width at half maximum. Values with the same superscript letters showed no significant difference in rows (*p* > 0.05).

		The Mean of Peak Interval Time (msec)				The Mean of Full Width at Half Maximum (msec)			
Participant	Trial	MGL	MGR	EMGL	EMGR	MGL	MGR	EMGL	EMGR
A	1	280 ± 23 ^a^	297 ± 64 ^a^	281 ± 33 ^a^	280 ± 24 ^a^	166 ± 16 ^a^	159 ± 17 ^a^	110 ± 22 ^b^	107 ± 17 ^b^
	2	303 ± 22 ^a^	303 ± 22 ^a^	305 ± 22 ^a^	302 ± 26 ^a^	173 ± 8 ^a^	173 ± 10 ^a^	109 ± 25 ^b^	104 ± 24 ^b^
	3	296 ± 27 ^a^	296 ± 29 ^a^	295 ± 39 ^a^	294 ± 35 ^a^	166 ± 13 ^a^	166 ± 17 ^a^	107 ± 22 ^b^	108 ± 21 ^b^
B	1	563 ± 48 ^a^	564 ± 50 ^a^	566 ± 51 ^a^	566 ± 61 ^a^	227 ± 30 ^a^	234 ± 30 ^a^	228 ± 35 ^a^	236 ± 38 ^a^
	2	312 ± 19 ^a^	312 ± 18 ^a^	312 ± 23 ^a^	311 ± 24 ^a^	159 ± 11 ^a^	161 ± 9 ^a^	109 ± 19 ^b^	114 ± 20 ^b^
	3	305 ± 23 ^a^	305 ± 22 ^a^	304 ± 31 ^a^	304 ± 27 ^a^	160 ± 10 ^a^	160 ± 9 ^a^	115 ± 21 ^b^	127 ± 22 ^b^
C	1	337 ± 19 ^a^	337 ± 19 ^a^	336 ± 28 ^a^	336 ± 32 ^a^	239 ± 27 ^a^	196 ± 18 ^b^	136 ± 32 ^c^	130 ± 30 ^c^
	2	323 ± 24 ^a^	323 ± 21 ^a^	323 ± 44 ^a^	321 ± 31 ^a^	240 ± 40 ^a^	198 ± 24 ^b^	134 ± 30 ^c^	135 ± 28 ^c^
	3	311 ± 21 ^a^	311 ± 18 ^a^	308 ± 39 ^a^	313 ± 40 ^a^	235 ± 30 ^a^	193 ± 19 ^b^	137 ± 24 ^c^	136 ± 29 ^c^

**Table 3:** MG sensor and EMG results during left and right movement.

		Left (%)				Right (%)			
Participant	Trial	MGL	MGR	EMGL	EMGR	MGL	MGR	EMGL	EMGR
A	1	66 ± 10	3.2 ± 0.7	61 ± 11	67 ± 17	3.4 ± 0.9	72 ± 19	57 ± 21	36 ± 23
	2	70 ± 8	4.4 ± 0.6	72 ± 15	76 ± 22	4.2 ± 1.1	85 ± 14	71 ± 21	42 ± 22
	3	72 ± 4	4.7 ± 0.3	68 ± 15	100 ± 22	4.9 ± 1.0	81 ± 9	79 ± 19	54 ± 33
B	1	56 ± 11	65 ± 17	74 ± 26	83 ± 18	49 ± 6	99 ± 0.3	71 ± 11	83 ± 18
	2	70 ± 13	82 ± 10	82 ± 28	82 ± 17	21 ± 11	40 ± 13	41 ± 18	56 ± 16
	3	59 ± 14	85 ± 13	57 ± 14	69 ± 13	43 ± 7	84 ± 7	57 ± 9	62 ± 13
C	1	86 ± 4	2.4 ± 0.2	49 ± 8	36 ± 6	70 ± 4	99 ± 0.2	65 ± 12	79 ± 22
	2	89 ± 2	2.4 ± 0.3	49 ± 8	39 ± 7	56 ± 1	83 ± 4	42 ± 6	53 ± 9
	3	91 ± 2	2.6 ± 0.2	45 ± 8	39 ± 7	54 ± 6	78 ± 5	34 ± 9	55 ± 10

## Data Availability

The data that support the findings of this study are available from the corresponding author (T.W.) upon reasonable request.
